# Association of novel anthropometric indices with prevalence of kidney stone disease: a population-based cross-sectional study

**DOI:** 10.1186/s40001-024-01743-5

**Published:** 2024-03-27

**Authors:** Xudong Hu, Xiang Li, Nan Ye, Zhenwen Zhou, Guangyuan Li, Fang Jiang

**Affiliations:** 1 Department of Urology, The First Affiliated Hospital of Anhui Medical University, 100 Huaihai Road, Hefei, 230000 Anhui China; 2 Department of Urology, Anhui Public Health Clinical Center, 100 Huaihai Road, Hefei, 230000 Anhui China; 3 Department of Urology, Kunshan Hospital of Traditional Chinese Medicine, Kunshan, 215300 Jiangsu China; 4 Department of Urology, Anqing First People’s Hospital of Anhui Province, 42 Xiaosu Road, Anqing, 246000 Anhui China

**Keywords:** A body shape index, Body roundness index, Cross-sectional study, Kidney stone disease, United States

## Abstract

**Background:**

The purpose of this study was to investigate the correlation between novel anthropometric indices, specifically the body shape index (ABSI) and body roundness index (BRI), and the prevalence of kidney stone disease (KSD) within the general population of the United States (U.S.).

**Methods:**

This study employed a cross-sectional analysis of participants in the National Health and Nutrition Examination Survey from 2007 to 2020. Various statistical methods, including multivariable logistic regression analysis, restricted cubic spline (RCS) plot curve, receiver operating characteristic (ROC) curves, and subgroup analysis, were utilized to examine the association between ABSI and BRI and the risk of KSD.

**Results:**

A total of 39,251 individuals were included in the study. First, the RCS plot presented that a linear positive association was found between ABSI and BRI and KSD risk. Second, the results of the multivariable logistic regression analysis revealed that, compared to the lowest quartile, the adjusted odds ratios (with 95% confidence intervals) for the prevalence of KSD across the quartiles of ASBI and BRI were 0.94 (0.67, 1.30), 1.55 (1.15, 2.10), and 1.74 (1.28, 2.35), respectively, in the fully adjusted model. Third, the ROC curve demonstrated that the area under the curve of ABSI, and BRI was significantly higher than traditional anthropometry or body composition measures, including BMI and waist circumference.

**Conclusions:**

The findings of our study indicate that the discriminant ability of ABSI and BRI for KSD is significantly superior to that of BMI and waist circumference. Consequently, ABSI and BRI have the potential to more accurately identify an individual’s risk of developing KSD in a clinical setting.

**Supplementary Information:**

The online version contains supplementary material available at 10.1186/s40001-024-01743-5.

## Introduction

Kidney stone disease (KSD) is characterized by the abnormal accumulation of crystalline substances, such as uric acid, calcium oxalate, and calcium phosphate, within the renal calyces, renal pelvis, and the junction of the renal pelvis and the ureter [1]. Meanwhile, KSD is a prevalent condition in urology treatment, affecting approximately 1–20% of the global population. This disease often leads to kidney colic, urinary system infection, and kidney function impairment, posing significant risks to public health [2]. There are approximately 5% of women and 12% of men in the United States who will develop KSD during the course of their lifetimes, and the prevalence of KSD has been rising in both sexes [3]. In addition, recent research indicates a rise in the 12-month cumulative incidence of KSD among Americans, increasing from 0.6% in 2005 to 0.9% in 2015 [4]. The financial burden associated with KSD is substantial, with an estimated expenditure of $2 billion in 2000, and projections suggest that by 2030, the cost of treating KSD will surpass $4.5 billion [5].

With the advancement of research into KSD, it has been found that it is closely related to sex, age, race, body mass index (BMI), hypertension, diabetes, smoking, and the metabolic syndrome [6]. The shift in dietary patterns resulting from improved living standards has led to a rise in the prevalence of overweight and obesity among individuals [7]. Investigations have demonstrated a positive correlation between BMI and the incidence of KSD, indicating that being overweight or obese heightens the susceptibility to this disease [8]. Previous studies have predominantly employed BMI as a primary measure for evaluating obesity. The problem is, however, that it does not accurately reflect actual obesity levels and the distribution of body fat on an individual basis. However, BMI does not adequately reflect the distribution of abdominal fat [9]. Hence, a range of innovative anthropometric measurements, such as waist-to-height ratio (WHtR), waist-to-hip ratio (WHR), body roundness index (BRI), visceral adiposity index (VAI), and a body shape index (ABSI), have been proposed to enhance the assessment of both visceral adipose quantity and abdominal obesity severity [10]. Among them, ABSI serves as a numerical index designed to evaluate body shape and fat distribution in individuals, incorporating waist circumference, height, and weight to derive the index value [11]. ABSI was developed with the intention of offering supplementary insights beyond BMI by specifically examining the distribution of adipose tissue. Research findings have indicated that individuals with elevated waist circumference and central obesity, characterized by excessive fat accumulation around the abdomen, may face an augmented susceptibility to various health ailments, including cardiovascular disease and type 2 diabetes [12–14]. Meanwhile, Thomas et al. put forth the body roundness index (BRI) as a prospective metric for assessing visceral adiposity tissue and overall body fat percentage [15]. Furthermore, the effectiveness of the body roundness index (BRI) as a dependable indicator of metabolic syndrome has been demonstrated in diverse populations and ethnic groups [16]. Wang J et al. discovered a positive correlation between the prevalence of kidney stone disease (KSD) and the visceral adiposity index (VAI), suggesting that a lower VAI is associated with a reduced risk of future kidney stone formation [17]. Moreover, Lee MR and colleagues also unveiled that waist-to-height ratio (WHtR), waist-to-hip ratio (WHR), and VAI were all linked to a higher prevalence of KSD and an increased likelihood of developing incident KSD. Consequently, these variables possess potential as prognostic indicators within the clinical domain for the emergence of KSD [18]. However, there is a lack of research on the correlation between ABSI, and BRI and prevalence of KSD in United States (U.S.) adults. Consequently, we undertook this cross-sectional study to evaluate the connection between ABSI, and BRI, and the susceptibility to KSD in the U.S., utilizing data derived from the National Health and Nutrition Examination Survey (NHANES) database. This endeavor aims to facilitate healthcare practitioners in their assessment of KSD risk. In addition, we also identified and compared the discriminating power of novel anthropometric measures as a KSD risk screening tool, calculating the best cutoff values for these measures to help healthcare professionals assess KSD risk.

## Materials and methods

### Study population

**Fig. 1:**
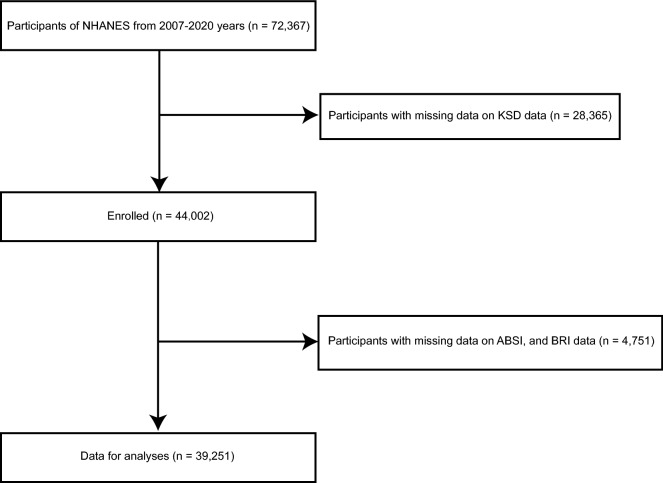
Study flow chart. NHANES, National Health and Nutrition Examination Surveys; ABSI, a body shape index; BRI, body roundness index; KSD, kidney stone disease

Based on a stratified multistage random sampling design, the NHANES is a nationally representative cross-sectional survey, which to reflect the civilian noninstitutionalized resident population information, conducted in the American. Data for this study were obtained from the NHANES surveys conducted between 2007 and 2020 year. In total, 72,367 patients were admitted, of whom 44,002 had KSD data available. Then, we excluded 4751 participants with missing the novel anthropometric indices, including a body shape index (ABSI), and body roundness index (BRI) data. Ultimately, we included 39,251 participants in our study (Fig. 1). Participants in the NHANES study signed informed consent forms and the protocols were approved by the National Center For Health Statistics Research Ethics Review Board [19]. The NHANES website (https://www.cdc.gov/nchs/nhanes/) provides information about the survey design, methods, population, and data.

### Anthropometric measurements

Using standardized techniques and equipment, experienced examiners measured basic anthropometric measurements at the mobile examination center, including body height (BH), weight (BW), and waist circumference (WC). Among them, WC was measured at the superior border of the iliac crests. The ABSI and BRI were calculated according to the previous published formulae [20] as follows:

$${\text{ABSI }} = \, {{{\text{WC }}\left( {{\text{cm}}} \right)} \mathord{\left/ {\vphantom {{{\text{WC }}\left( {{\text{cm}}} \right)} {{\text{BMI}}^{{{2}/{3}}} \left( {{\text{kg}}/{\text{m}}^{{2}} } \right)}}} \right. \kern-0pt} {{\text{BMI}}^{{{2}/{3}}} \left( {{\text{kg}}/{\text{m}}^{{2}} } \right)}} \, \times {\text{ BH}}^{{{1}/{2}}} \left( {\text{m}} \right)$$

$${\text{BRI}}=364.2-365.5\times \sqrt{1-{\left(\frac{\frac{WC\left(m\right)}{2\pi }}{0.5\times BH\left(m\right)}\right)}^{2}}$$

### KSD measurement

A questionnaire called KIQ026 was completed by all participants at baseline. The question in the questionnaire was “Have you ever had a kidney stone?”. A history of kidney stones was considered to be present in participants who replied "Yes". Those participants who said “no” to the question of whether they had had a kidney stone were assumed to have not had one. NHANES CAPI system with built-in consistency checks ensured the quality and effectiveness of this question. More details are documented in the NHANES Laboratory/Medical Technician Procedures Manual.

### Covariates

The following covariates were included in the study: race/ethnicity (Other Hispanic, Non-Hispanic White, Mexican American, Non-Hispanic Black, and Other Race—Including Multi-Racial), age, sex, family poverty income ratio (PIR), marital status (having a partner, no partner, and unmarried), education level (less than high school, high school, and more than high school), smoke status (no, former, and now), drinking status (no, former, mild, moderate, and heavy), the complication of hypertension, and diabetes mellitus (DM), BMI, WC, ABSI, BRI, mean energy intake, total water intake, urine albumin, total cholesterol (TC), urine creatinine, fast glucose (FBG), uric acid (UA), triglyceride (TG), serum creatinine (Scr), urea albumin creatinine ratio (uACR), blood urea nitrogen (BUN), high-density lipoprotein-cholesterol (HDL-C), and estimated glomerular filtration rate (eGFR). For more information about the variables in this study, please refer to www.cdc.gov/nchs/nhanes/.

### Statistical analysis

In this study, all statistical analyses were conducted using R version 4.2.3 (R Foundation for Statistical Computing, Vienna, Austria) and SPSS version 23.0 (SPSS Inc., Chicago, IL, USA). To qualify as statistically significant, *P* value < 0.05 had to be met. All estimates were calculated by accounting for NHANES sample weights. The characteristics of the participants were subclassified based on ABSI quartiles (Q1: 0.0576–0.078; Q2: 0.079–0.082; Q3: 0.083–0.085; and Q4: 0.086–0.112), BRI quartiles (Q1: 1.049–3.903; Q2: 3.904–5.205; Q3: 5.206–6.820; and Q4: 6.821–23.483), BMI quartiles (Q1: 15.02–24.50; Q2: 24.51–28.20; Q3: 28.21–32.90; and Q4: 32.91–82.00), and WC quartiles (Q1: 61.10–88.30; Q2: 88.31–98.50; Q3: 98.51–109.75; and Q4: 109.76–178.00). Variables that are continuous are expressed in terms of means (standard deviations, SDs) and variables that are categorical are expressed in terms of numbers (%). We used weighted Student’s *t* test or one-way ANOVA methods (continuous variables) and weighted Chi-square tests method (categorical variables) to calculate differences between groups. An analysis of multivariate logistic regression was used to investigate the association of ABSI, BRI, BMI, and WC with KSD risk. First, model 1 was adjusted for sex and age. Second, model 2 was adjusted for model 1 variables plus education level, race/ethnicity, marital status, smoke status, family PIR, drink status, the complication of DM, and hypertension. Finally, model 3 was adjusted for model 2 variables plus total water intake, FBG, TC, urine creatinine, UA, HDL-C, mean energy intake, BUN, urine albumin, Scr, uACR, TG, and eGFR, as our final model.

## Results

### Baseline characteristics

We computed that the number of participants in this research may be representative of the total population of 216,538,371 in the United States. Table 1 shows the baseline characteristics of the research participants in detail and the incidence of KSD in this group was 10.1%. There is a significant difference in age, marital status, sex, the complication of hypertension, smoker, race/ethnicity, work activity, BMI, the complication of DM, urine albumin, waist circumference, alcohol user, FBG, eGFR, TG, BUN, Scr, HDL, UA, urine creatinine, uACR, ABSI, and BRI among non-KSD group, and KSD group.

**Table 1:** Demographic characteristics of the study participants

Variables	Overall (*n* = 39,251)	Non-KSD (*n* = 35,560)	KSD (*n *= 3691)	*P* value
Age, years	47.33 ± 0.21	46.69 ± 0.21	53.20 ± 0.29	< 0.001
Sex, %				< 0.001
Male	19,125 (48.7%)	17,082 (43.5%)	2043 (5.2%)	
Female	20,126 (51.3%)	18,478 (47.1%)	1648 (4.2%)	
Race/ethnicity, %				< 0.001
Mexican American	5654 (14.4%)	5197 (13.2%)	457 (1.2%)	
Other Hispanic	4114 (10.5%)	3682 (9.4%)	432 (1.1%)	
Non-Hispanic Black	8815 (22.5%)	8299 (21.1%)	516 (1.3%)	
Non-Hispanic White	15,455 (39.4%)	13,529 (34.5%)	1926 (4.9%)	
Other race	5213 (13.3%)	4853 (12.4%)	360 (0.9%)	
Family PIR	3.00 ± 0.03	2.99 ± 0.03	3.04 ± 0.05	0.319
Education level, %				0.946
Less than high school	9091 (23.2%)	8208 (20.9%)	883 (2.2%)	
High school	6432 (16.4%)	5850 (16.5%)	582 (1.5%)	
More than high school	23,728 (60.5%)	21,502 (54.8%)	2226 (5.7%)	
Marital status, %				< 0.001
Having a partner	23,297 (59.4%)	20,963 (53.4%)	2334 (5.9%)	
No partner	8596 (21.9%)	7635 (19.5%)	961 (2.4%)	
Unmarried	7358 (18.7%)	6962 (17.7%)	396 (1.0%)	
Hypertension, %				< 0.001
No	23,020 (58.6%)	21,359 (54.4%)	1661 (4.2%)	
Yes	16,231 (41.4%)	14,201 (36.2%)	2030 (5.2%)	
DM, %				< 0.001
No	31,731 (80.8%)	29,140 (74.2%)	2591 (6.6%)	
Yes	7520 (19.2%)	6420 (16.4%)	1100 (2.8%)	
Smoker, %				< 0.001
No	22,135 (56.4%)	20,282 (51.7%)	1853 (4.7%)	
Former	9244 (23.6%)	8135 (20.7%)	1109 (2.8%)	
Now	7872 (20.1%)	7143 (18.2%)	729 (1.9%)	
Alcohol user, %				< 0.001
No	5539 (14.1%)	5051 (12.9%)	488 (13.2%)	
Former	4809 (12.3%)	4223 (10.8%)	586 (1.5%)	
Mild	14,304 (36.4%)	12,830 (32.7%)	1474 (3.8%)	
Moderate	6526 (16.6%)	5986 (15.3%)	540 (1.4%)	
Heavy	8073 (20.6%)	7470 (19.0%)	603 (1.5%)	
Work activity, %				0.003
No	22,530 (57.4%)	20,454 (52.1%)	2076 (5.3%)	
Moderate	8487 (21.6%)	7722 (19.7%)	765 (1.9%)	
Both	6579 (16.8%)	5905 (15.0%)	674 (1.7%)	
Vigorous	1665 (4.2%)	1479 (3.8%)	176 (0.4%)	
BMI, kg/m^2^	29.11 ± 0.08	28.94 ± 0.08	30.63 ± 0.16	< 0.001
Waist circumference, cm	99.47 ± 0.20	98.91 ± 0.20	104.62 ± 0.41	< 0.001
Mean energy	2094.76 ± 6.43	2097.29 ± 6.51	2071.60 ± 18.65	0.173
Intake, kcal/day				
Total water	2252.63 ± 23.50	2260.92 ± 23.84	2176.68 ± 47.10	0.064
Drank, gm				
FBG, mg/dL	106.61 ± 0.21	105.91 ± 0.22	112.95 ± 0.79	< 0.0001
TC, mg/dL	192.49 ± 0.45	192.58 ± 0.44	191.70 ± 1.15	0.415
TG, mg/dL	122.94 ± 0.87	121.61 ± 0.88	135.07 ± 3.23	< 0.0001
HDL, mg/dL	53.61 ± 0.19	53.95 ± 0.20	50.49 ± 0.38	< 0.0001
BUN, mg/dL	13.83 ± 0.06	13.70 ± 0.06	15.01 ± 0.14	< 0.0001
Scr, mg/dL	0.88 ± 0.00	0.87 ± 0.00	0.93 ± 0.01	< 0.0001
UA, mg/dL	5.40 ± 0.01	5.38 ± 0.01	5.59 ± 0.03	< 0.0001
eGFR, ml/min/1.73m^2^	94.95 ± 0.29	95.66 ± 0.30	88.36 ± 0.41	< 0.0001
Urine albumin, mg/L	34.90 ± 1.47	32.38 ± 1.34	58.00 ± 6.93	< 0.001
Urine creatinine, mg/dL	122.14 ± 0.80	121.66 ± 0.85	126.54 ± 1.96	0.022
uACR, mg/g	35.17 ± 1.78	32.86 ± 1.84	56.30 ± 5.59	< 0.0001
ABSI	0.081 ± 0.01	0.081 ± 0.01	0.083 ± 0.001	< 0.001
BRI	5.43 ± 0.03	5.36 ± 0.03	6.11 ± 0.06	< 0.001

DM, diabetes mellitus; BMI, body mass index; FBG, fast glucose; TC, total cholesterol; TG, triglycerides; HDL-cholesterol, high density lipoprotein–cholesterol; BUN, blood urea nitrogen; UA, uric acid; Scr, serum creatinine; eGFR, estimated glomerular filtration rate; uACR, urea albumin creatinine ratio; ABSI, a body shape index; BRI, body roundness index

### Association between ABSI, and BRI and KSD

**Fig. 2:**
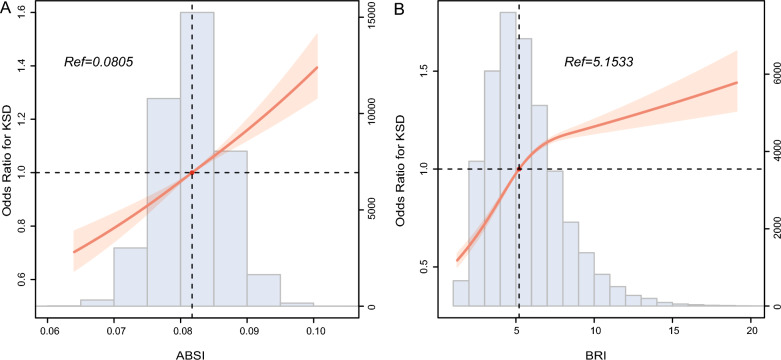
RCS curve of the association of **A** ABSI, and **B** BRI with KSD. RCS, restricted cubic spline; ABSI, a body shape index; BRI, body roundness index; KSD, kidney stone disease

**Fig. 3:**
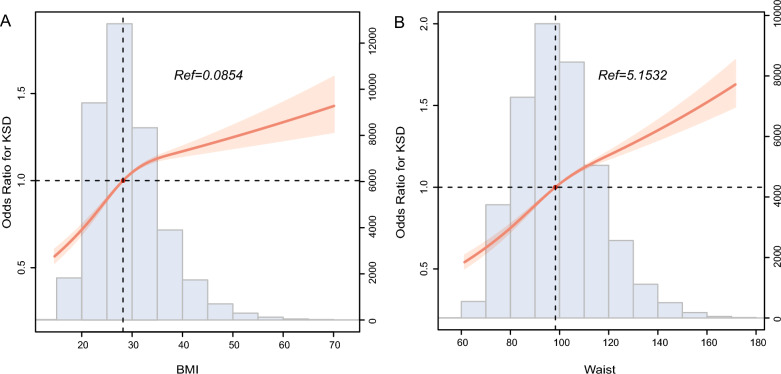
RCS curve of the association of **A** BMI, and **B** waist circumference with KSD. RCS, restricted cubic spline; BMI, body mass index; KSD, kidney stone disease

The RCS plot illustrated that ABSI, BRI, BMI, and waist circumference were positively correlated with risk of KSD (*P* for nonlinearity > 0.05, Figs. 2A, B, 3A, B). In addition, Table 2 shows the findings of multivariate logistic regression analysis for the relationship between ABSI, and BRI and prevalence of KSD. After adjusting for confounding factors, compared with the lowest quartiles (Q1) of ABSI, and BRI, the odds ratios (ORs) with 95% confidence intervals (CIs) for KSD across the quartiles were 0.968 (0.575, 1.630), 1.092 (0.584, 2.044), and 1.453 (0.625, 3.376), as well as 1.196 (0.717, 1.994), 1.148 (0.603, 2.187), and 1.761 (0.690, 4.492), respectively. In addition, we also presented the findings of multivariate logistic regression analysis for the association of BMI, WC with prevalence of KSD (Table 3).

**Table 2:** Associations of ABSI, and BRI with risk of KSD

	Model 1		Model 2		Model 3	
	OR (95%CI)	*P* for trend	OR (95%CI)	*P* for trend	OR (95%CI)	*P* for trend
ABSI		< 0.001		< 0.001		< 0.001
0.0576–0.078	1.00		1.00		1.00	
0.079–0.082	1.20 (1.07, 1.34) **		1.09 (0.97, 1.22)		1.08 (0.97, 1.22)	
0.083–0.085	1.43 (1.27, 1.61) **		1.23 (1.09, 1.38) **		1.22 (1.08, 1.37) **	
0.086– 0.112	1.60 (1.42, 1.80) ***		1.28 (1.13, 1.45) ***		1.28 (1.12, 1.45) ***	
BRI		< 0.001		< 0.001		< 0.001
1.049–3.903	1.00		1.00		1.00	
3.904–5.205	1.39 (1.24, 1.56) ***		1.34 (1.19, 1.50) ***		1.31 (1.16, 1.47) ***	
5.206–6.820	1.77 (1.58, 1.97) ***		1.64 (1.46, 1.84) ***		1.58 (1.40, 1.79) ***	
6.821–23.483	2.12 (1.90, 2.37) ***		1.82 (1.62, 2.05) ***		1.74 (1.53, 1.97) ***	

ABSI, a body shape index; BRI, body roundness index; KSD, kidney stone disease; OR, odds ratio; CI, confidence interval

Model 1: age, and sex

Model 2: model 1 variables plus race/ethnicity, education level, marital status, family poverty-income ratio, hypertension, diabetes mellitus, smoker, alcohol user

Model 3 was adjusted for model 2 variables plus mean energy intake, total water intake, fast glucose, total cholesterol, triglyceride, high-density lipoprotein–cholesterol, urine creatinine, urine albumin, urea albumin creatinine ratio, blood urea nitrogen, uric acid, serum creatinine, and estimated glomerular filtration rate

**P < 0.01, ***P < 0.001

**Table 3:** Associations of BMI, and waist circumference with the risk of KSD

	Model 1		Model 2		Model 3	
	OR (95%CI)	*P* for trend	OR (95%CI)	*P* for trend	OR (95%CI)	*P* for trend
BMI		< 0.001		< 0.001		< 0.001
15.02–24.50	1.00		1.00		1.00	
24.51–28.20	1.29 (1.16, 1.43)		1.24 (1.11, 1.38) ***		1.21 (1.08, 1.35) **	
28.21–32.90	1.54 (1.39, 1.71)		1.44 (1.29, 1.60) ***		1.37 (1.22, 1.53) ***	
32.91–82.00	1.85 (1.67, 2.04)		1.65 (1.48, 1.84) ***		1.54 (1.37, 1.74) ***	
Waist circumference		< 0.001		< 0.001		< 0.001
61.10–88.30	1.00		1.00		1.00	
88.31–98.50	1.25 (1.12, 1.40) **		1.19 (1.06, 1.34) **		1.16 (1.03, 1.30) **	
98.51–109.75	1.66 (1.49, 1.85) ***		1.50 (1.34, 1.68) ***		1.43 (1.27, 1.61) ***	
109.76–178.00	1.98 (1.78, 2.20) ***		1.67 (1.49, 1.87) ***		1.57 (1.39, 1.77) ***	

BMI, body mass index; KSD, kidney stone disease; OR, odds ratio; CI, confidence interval

Model 1: age, and sex

Model 2: model 1 variables plus race/ethnicity, education level, marital status, family poverty-income ratio, hypertension, diabetes mellitus, smoker, alcohol user

Model 3 was adjusted for model 2 variables plus mean energy intake, total water intake, fast glucose, total cholesterol, triglyceride, high-density lipoprotein–cholesterol, urine creatinine, urine albumin, urea albumin creatinine ratio, blood urea nitrogen, uric acid, serum creatinine, and estimated glomerular filtration rate

**P < 0.01, ***P < 0.001

### Discrimination ability of different anthropometric measures

**Fig. 4:**
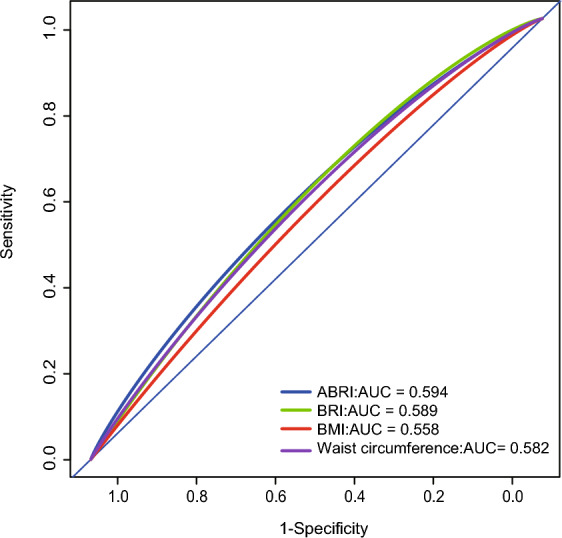
Receiver operating characteristic curves of ABSI, BRI, BMI, and waist circumference to identify subjects with KSD. ABSI, a body shape index; BRI, body roundness index; BMI, body mass index; KSD, kidney stone disease

We performed ROC curve to evaluate the abilities of two novel anthropometric measures in discriminating individuals with KSD. The ROC curve analysis showed that the discriminant ability of ABSI, and BRI for KSD was significantly higher than BMI, and waist circumference (Fig. 4). The area under curves of ABSI, BRI, BMI, and WC were 0.594, 0.589, 0.558, and 0.582, respectively.

### Subgroup analyses

Subgroup analysis, stratified by age, sex, hypertension, and DM were undertaken to explore the association between ABSI, BRI, BMI, and WC with KSD risk. The stratified analysis revealed that ABSI with risk of KSD was observed in participants who older than 60 years, without or with hypertension, male or female, and with DM (Additional file 1: Fig. S1, and Additional file 5: Table S1). The positive liner association of BRI with KSD was found among participants in all ages and was male or female, without or with hypertension, and without or with DM (Additional file 2: Fig. S2, and Additional file 6: Table S2). In addition, we further performed subgroup analysis to examine the correlation between BMI, and WC with KSD risk (Additional file 7: Table S3, and Additional file 7: Table S4; Additional file 3: Fig. S3, and Additional file 4: Fig. S4). Associations of BMI and waist circumference and prevalence of KSD were consistent with BRI in different subgroups.

## Discussion

There exist two prevalent metrics for assessing body shape, namely, BMI and WC. Nevertheless, BMI encounters a limitation in its inability to discern between fat mass and fat-free mass, whereas WC exhibits a bias against individuals of varying sizes, thereby diminishing its reliability [21–23]. Consequently, ABSI and BRI have emerged as novel anthropometric approaches to gauge both the extent of abdominal obesity and the quantity of visceral adipose tissue in conjunction with BMI and waist circumference. This study represents the initial investigation into the correlation between the recently developed anthropometric indices, ABSI and BRI, and the prevalence of KSD within the broader U.S. population. Our findings indicate a positive association between ABSI and BRI and the prevalence of KSD. Specifically, individuals with higher ABSI and BRI values exhibited a greater likelihood of experiencing KSD compared to those with lower ABSI and BRI values. Furthermore, when comparing these novel anthropometric indicators with traditional measures such as BMI and waist circumference, we observed a significantly higher predictive capacity of ABSI and BRI in discerning the likelihood of developing KSD. Based on our research findings, it is suggested that ABSI and BRI could play a crucial role in the management of risk associated with KSD. Currently, there is a dearth of studies investigating the correlation between ABSI and the prevalence of KSD. Nevertheless, prior research has established a strong link between obesity and the likelihood of developing KSD [24]. For instance, Ye Z et al. demonstrated that overweight or obesity, in conjunction with an unhealthy metabolic status, significantly heightens the risk of KSD within the general population of the U.S. [25]. Furthermore, Yuan S. et al. employed a Mendelian randomization analysis to propose a causal relationship between a high BMI and an augmented risk of KSD [26]. Similarly, Rahman IA et al. demonstrated an association between hypertension, DM, obesity, dyslipidemia, and an elevated likelihood of developing KSD [27]. In addition, when compared to the control group, patients with nephrolithiasis had significantly higher visceral adiposity indexes (VAI) [28]. Consequently, our hypothesis posits that the rise in ABSI and BRI among participants correlates with an increased prevalence of these conditions, ultimately resulting in a higher incidence of KSD. Lee MR and colleagues discovered that BRI exhibited a significant correlation with an increased prevalence of KSD and the emergence of incident KSD within the Chinese Taiwan population, thus potentially serving as predictive indicators for KSD development in clinical settings [18]. Nevertheless, investigations exploring the association between BRI and KSD in other populations remain lacking. Huang H. et al. also discovered that perirenal fat thickness is a noteworthy predictive factor for the development and recurrence of KSD in a Chinese population, suggesting its potential utility in risk stratification during follow-up periods [29]. Consequently, reducing the prevalence of obesity, particularly abdominal obesity, may have a crucial role in preventing KSD. Furthermore, ABSI and BRI exhibit superior discriminant capabilities in predicting KSD compared to BMI and waist circumference. Therefore, we speculated that abdominal obesity is more closely associated with the occurrence of KSD.

Utilizing a substantial sample population from the NHANES database spanning from 2007 to 2020, the present study's outcomes contribute supplementary evidence supporting the utilization of ABSI and BRI as tools for identifying populations susceptible to KSD in primary prevention. Nevertheless, it is imperative to acknowledge the limitations inherent in our study. First, due to its cross-sectional design and the characteristics of the included population, this investigation is unable to establish a temporal causal relationship between ABSI, BRI, and the prevalence of KSD. Second, our study employed rigorous control measures for influential confounders, including kidney function and total water intake, during the implementation of our multivariable logistic regression analysis and RCS model to assess the prevalence of KSD. Nonetheless, it is important to acknowledge the potential existence of other unadjusted confounding factors. Third, due to constraints imposed by the NHANES database, we were unable to access radiological examinations of the patients (kidney–ureter–bladder radiography, ultrasound, computed tomography), stone analysis reports, and hospitalization reports from 2007–2020 years. Fourth, in the subgroup analysis, there were differences in the number of KSD and non-KSD groups that may affect this relationship between ABSI, as well as BRI and KSD. Finally, whether the conclusion in the present study based on U.S. participants could be applicable to other populations need to be further explored in the future work.

## Conclusion

In summary, the study found a positive correlation between the ABSI, BRI, BMI, and waist circumference and the risk of KSD. Furthermore, the discriminant ability of ABSI and BRI in predicting KSD was significantly superior to that of BMI and waist circumference. More attention should be paid to anthropometric indices, especially novel anthropometric indices, to better prevent and treat of KSD.

### Supplementary Information

**Additional file 1: Figure S1.** RCS curve of the association of ABSI with KSD stratified by **A** age, **B** sex; **C** hypertension, and **D** DM. RCS, restricted cubic spline; ABSI, a body shape index; KSD, kidney stone disease; DM, diabetes mellitus

**Additional file 2: Figure S2.** RCS curve of the association of BRI with KSD stratified by **A** age, **B** sex; **C** hypertension, and **D** DM. RCS, restricted cubic spline; BRI, body roundness index; KSD, kidney stone disease; DM, diabetes mellitus.

**Additional file 3: Figure S3.** RCS curve of the association of BMI with KSD stratified by **A** age, **B** sex; **C** hypertension, and **D** DM. RCS, restricted cubic spline; BMI, body mass index; KSD, kidney stone disease; DM, diabetes mellitus.

**Additional file 4: Figure S4.** RCS curve of the association of waist circumference with KSD stratified by **A** age, **B** sex; **C** hypertension, and **D** DM. RCS, restricted cubic spline; KSD, kidney stone disease; DM, diabetes mellitus.

**Additional file 5: Table S1.** Subgroups analysis for the associations of ABSI with prevalence of KSD.

**Additional file 6: Table S2.** Subgroups analysis for the associations of BRI with prevalence of KSD.

**Additional file 7: Table S3.** Subgroups analysis for the associations of BMI with prevalence of KSD.

**Additional file 8: Table S4.** Subgroups analysis for the associations of waist circumference with prevalence of KSD.

## Data Availability

Survey data are available for data consumers and researchers all across the globe on the internet (https://www.cdc.gov/nchs/nhanes/).
