# Supplementary figures and images for: Association of novel anthropometric indices with prevalence of kidney stone disease: a population-based cross-sectional study
Source: Eur J Med Res. 2024 Mar 27;29:204. doi: 10.1186/s40001-024-01743-5 (PMC10967179; doi:10.1186/s40001-024-01743-5)

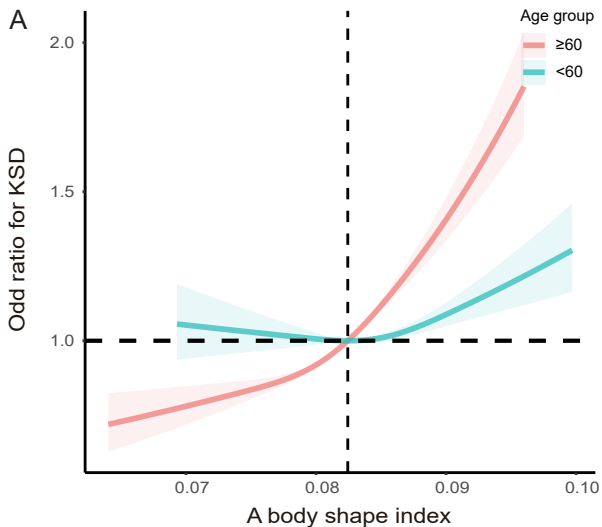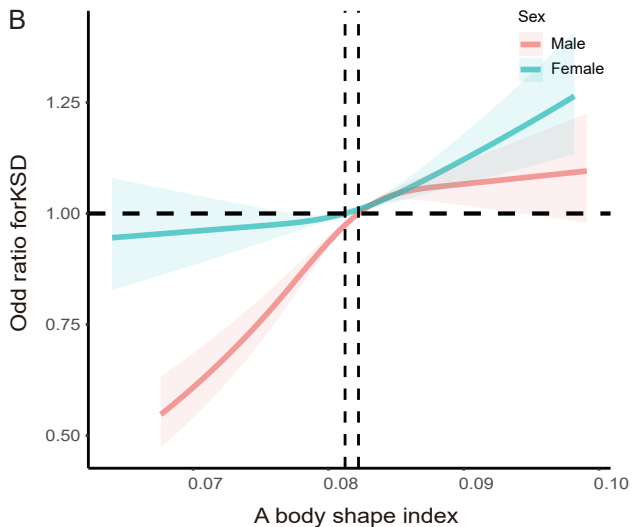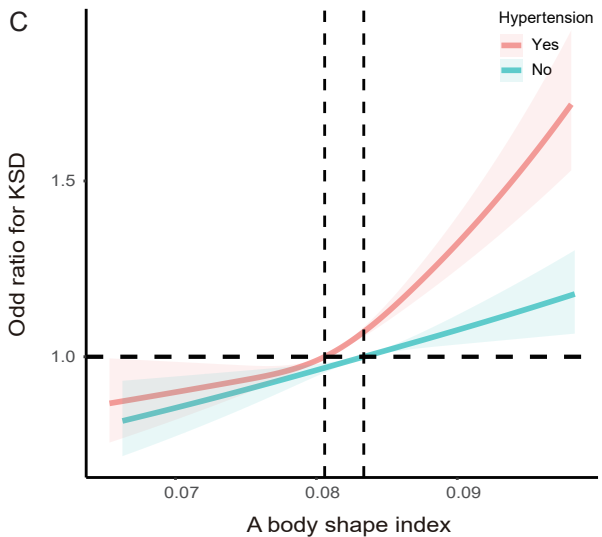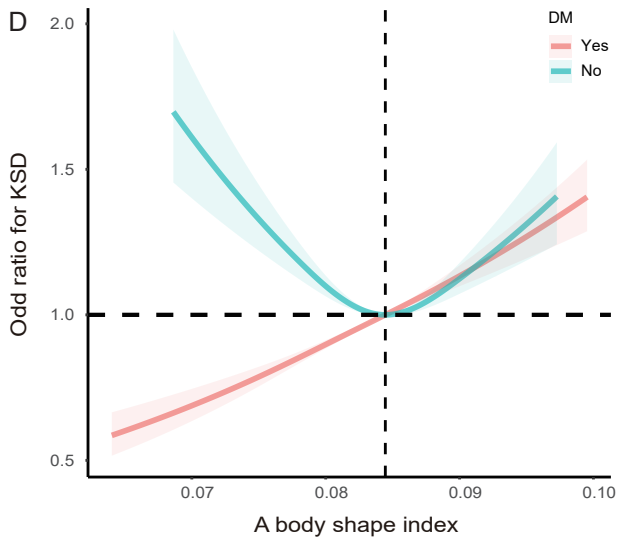

Supplement: Supplementary file 1 — Additional file 1: Figure S1. RCS curve of the association of ABSI with KSD stratified by A age, B sex; C hypertension, and D DM. RCS, restricted cubic spline; ABSI, a body shape index; KSD, kidney stone disease; DM, diabetes mellitus [file 40001_2024_1743_MOESM1_ESM.pdf]

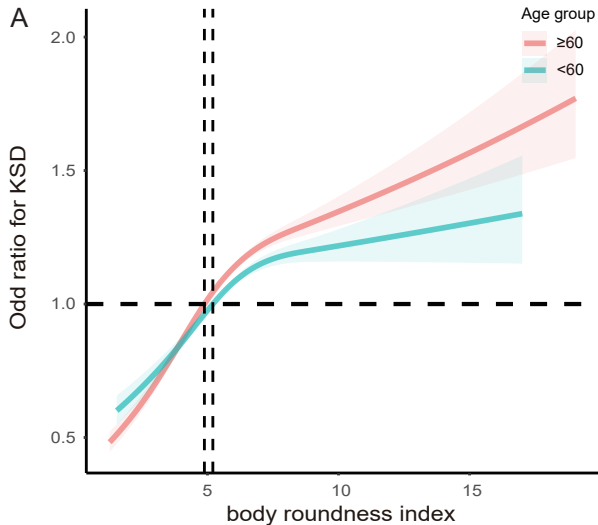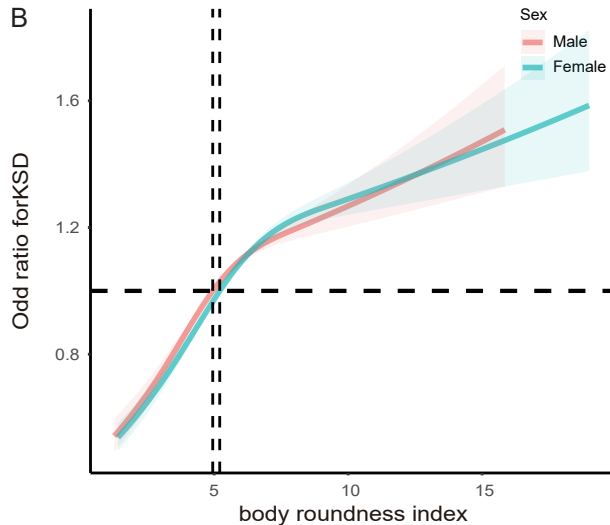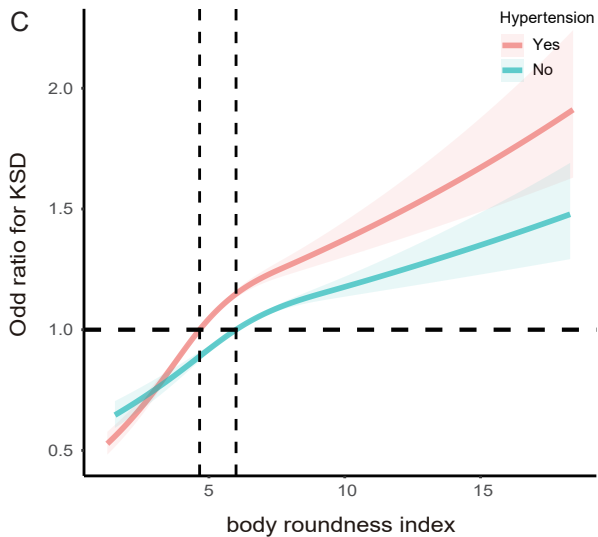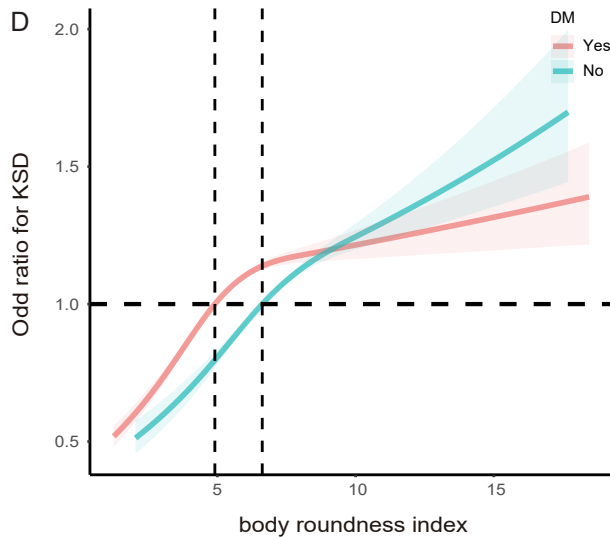

Supplement: Supplementary file 2 — Additional file 2: Figure S2. RCS curve of the association of BRI with KSD stratified by A age, B sex; C hypertension, and D DM. RCS, restricted cubic spline; BRI, body roundness index; KSD, kidney stone disease; DM, diabetes mellitus. [file 40001_2024_1743_MOESM2_ESM.pdf]

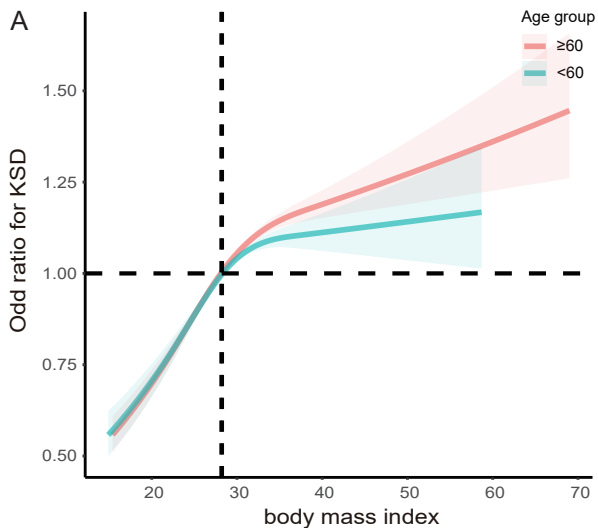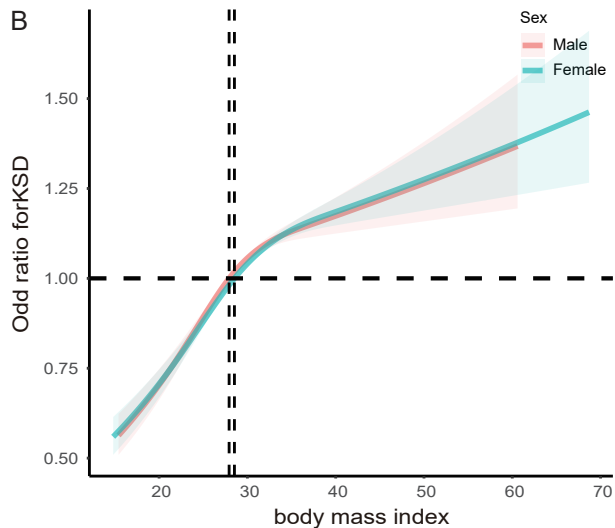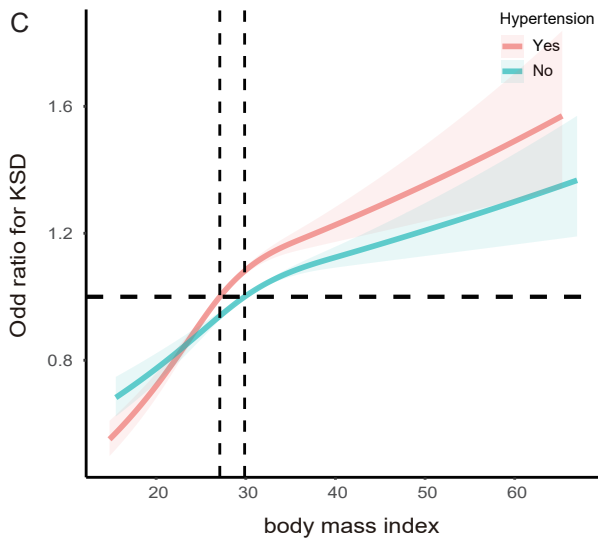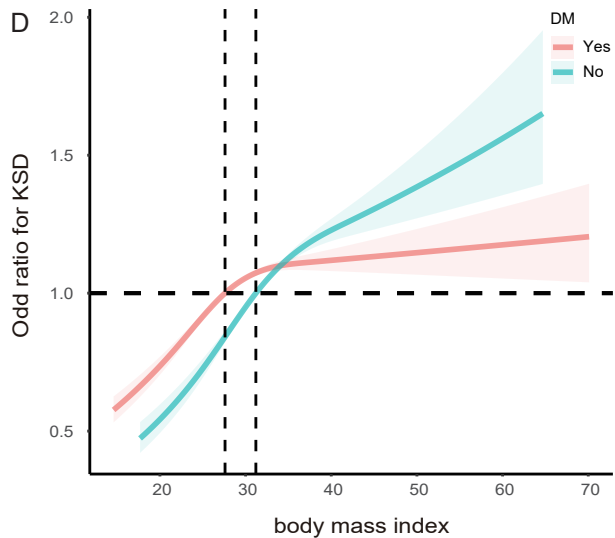

Supplement: Supplementary file 3 — Additional file 3: Figure S3. RCS curve of the association of BMI with KSD stratified by A age, B sex; C hypertension, and D DM. RCS, restricted cubic spline; BMI, body mass index; KSD, kidney stone disease; DM, diabetes mellitus. [file 40001_2024_1743_MOESM3_ESM.pdf]

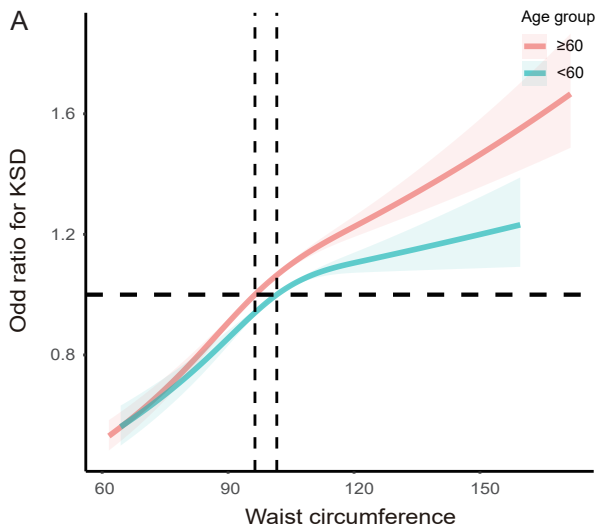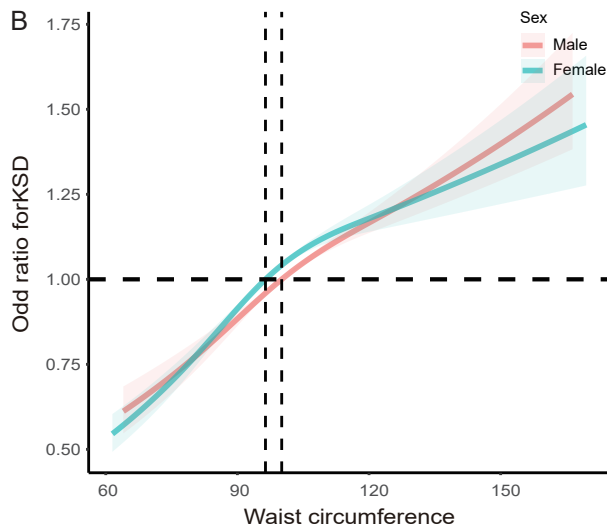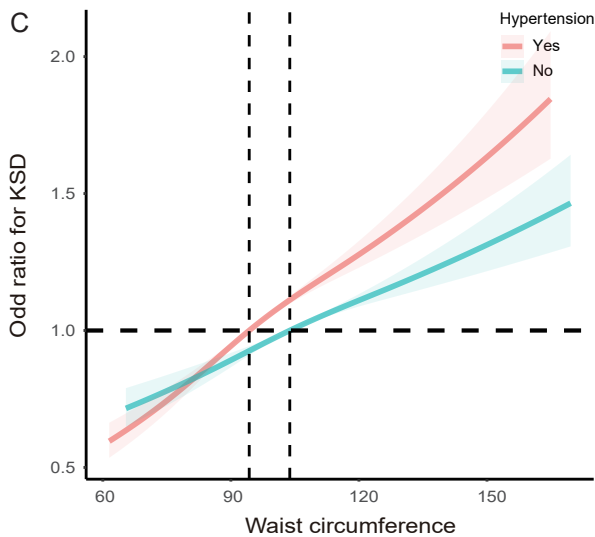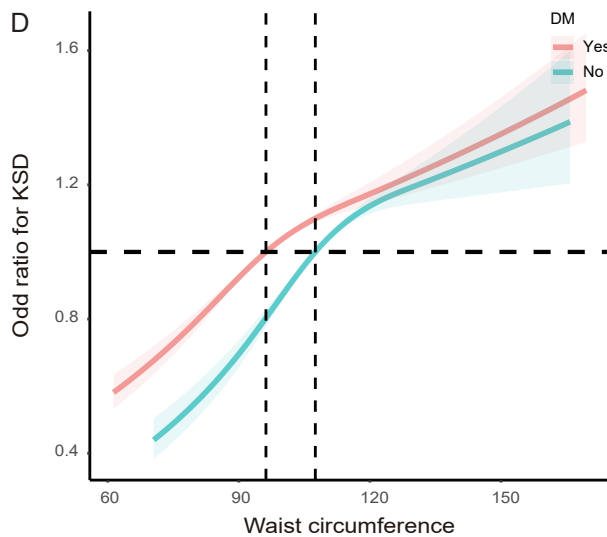

Supplement: Supplementary file 4 — Additional file 4: Figure S4. RCS curve of the association of waist circumference with KSD stratified by A age, B sex; C hypertension, and D DM. RCS, restricted cubic spline; KSD, kidney stone disease; DM, diabetes mellitus. [file 40001_2024_1743_MOESM4_ESM.pdf]
