# Supplementary material for: Association of novel anthropometric indices with prevalence of kidney stone disease: a population-based cross-sectional study
Source: Eur J Med Res. 2024 Mar 27;29:204. doi: 10.1186/s40001-024-01743-5 (PMC10967179; doi:10.1186/s40001-024-01743-5)
Supplement: Supplementary file 5 — Additional file 5: Table S1. Subgroups analysis for the associations of ABSI with prevalence of KSD. [file 40001_2024_1743_MOESM5_ESM.docx]

| **Supplementary Table 1.** Subgroups analysis for the associations of ABSI with the prevalence of KSD. | | | | | | |
| --- | --- | --- | --- | --- | --- | --- |
|  | Q1 | Q2 | Q3 | Q4 | *P* for trend | *P* for interaction |
|  | OR (95%CI) | OR (95%CI) | OR (95%CI) | OR (95%CI) |  |  |
| Age |  |  |  |  |  | 0.004 |
| < 60 | 1.00 | 1.11 (0.98,1.27) | 1.36 (1.18, 1.56) | 1.51 (1.29, 1.76) | <0.001 |  |
| ≥ 60 | 1.00 | 1.00 (0.77, 1.28) | 0.95 (0.74, 1.21) | 1.01 (0.80, 1.28) | 0.766 |  |
| Sex |  |  |  |  |  | <0.001 |
| Male | 1.00 | 1.15 (0.95, 1.39) | 1.33 (1.10, 1.62) ** | 1.30 (1.05, 1.60) * | 0.013 |  |
| Female | 1.00 | 1.05 (0.91, 1.22) | 1.07 (0.91, 1.26) | 1.16 (0.99, 1.37) | 0.068 |  |
| Hypertension | |  |  |  |  | <0.001 |
| No | 1.00 | 1.04 (0.89, 1.21) | 1.21 (1.02, 1.42) * | 1.42 (1.19, 1.69) *** | <0.001 |  |
| Yes | 1.00 | 1.06 (0.88, 1.27) | 1.14 (0.95, 1.36) | 1.12 (0.93, 1.35) | 0.189 |  |
| DM |  |  |  |  |  | <0.001 |
| No | 1.00 | 1.11 (0.97, 1.26) | 1.28 (1.12, 1.47) *** | 1.45 (1.26, 1.68) *** | <0.001 |  |
| Yes | 1.00 | 0.87 (0.66, 1.14) | 0.86 (0.65, 1.12) | 0.80 (0.61, 1.05) | 0.134 |  |

Abbreviations: ABSI, a body shape index; AAC, abdominal aortic calcification; DM, diabetes mellitus; Q1, 0.068–0.080; Q2, 0.081–0.083; Q3, 0.084–0.086; Q4, 0.087–0.108; OR, odd ratio; CI, confidence interval; Analysis was adjusted for age, sex, race/ethnicity, education level, marital status, family poverty-income ratio, hypertension, DM, smoker, alcohol user, coronary heart disease, congestive heart failure, angina pectoris, heart attack, and stroke, systolic blood pressure, diastolic blood pressure, mean energy intake, dietary calcium intake, dietary phosphorus intake, hemoglobin, fast glucose, fast insulin, glycohemoglobin, alkaline phosphatase, total bilirubin, serum phosphorus, and calcium, total cholesterol, triglyceride, high-density lipoprotein-cholesterol, blood urea nitrogen, uric acid, serum creatinine, estimated glomerular filtration rate.
